# Case report: Acute ischemic stroke caused by intracranial artery dissection in a patient with skull fractures

**DOI:** 10.3389/fneur.2022.963396

**Published:** 2022-10-24

**Authors:** Bo Zheng, XiaoLan Luo, JiangHao Zhou, XueQiong Huang, MaoXia Li, Hui Zheng, YiPing Yuan, Jian Wang

**Affiliations:** ^1^Department of Neurology, Yaan People's Hospital, Yaan, China; ^2^Department of Oncology, Yaan People's Hospital, Yaan, China; ^3^Department of Neurology, Yaan Second People's Hospital, Yaan, China

**Keywords:** skull fractures, blunt cerebrovascular injuries, intracranial artery dissection, middle cerebral artery dissection, acute ischemic stroke

## Abstract

The intracranial artery dissection (IAD) is an uncommon but life-threatening disease. The IAD would develop a significant cerebral infarction due to unrecognized contrecoup brain injury. We report a 53-year-old man fell to develop blunt cerebrovascular injuries (BCVIs) more than 2 months ago. During his rehabilitation, he often had a transient left headache and underwent short-term right limb weakness twice, but he did not care. He was hospitalized again because of suffering right limb weakness for more than 4 h. The brain computed tomography angiography (CTA) showed subtotal occlusion of the left middle cerebral artery M1 segment, and the vascular morphology displayed the IAD. The patient was then treated with balloon dilation and a self-expanding stent. This case highlights that IAD may show delayed onset with no initial typical symptom. By early detecting of abnormal signs and symptoms, serious traumatic brain injury may be avoided.

## Introduction

The incidence of blunt cerebrovascular injuries (BCVIs) accounted for ~1% of patients with blunt trauma after bone injuries (1), and it was even up to 9.2% after severe traumatic brain injury (TBI). The main risk factors may be high-velocity lesions and injuries near cervical arteries (2). The intracranial artery dissection (IAD) in the BCVIs is very rare, and this has long been considered a neglectful disease. It may be diagnosed by imaging examination only after clinically recognizable neurological symptoms. So, there is little research about the related potential risk for IAD in BCVIs. Our case manifested that unusual symptoms in convalescence may result in IAD after severe TBI. Increased awareness of this possibility was crucial to facilitate early recognition and initiate early intervention to prevent further complications, such as acute ischemic stroke (AIS).

## Case presentation

**Figure 1:**
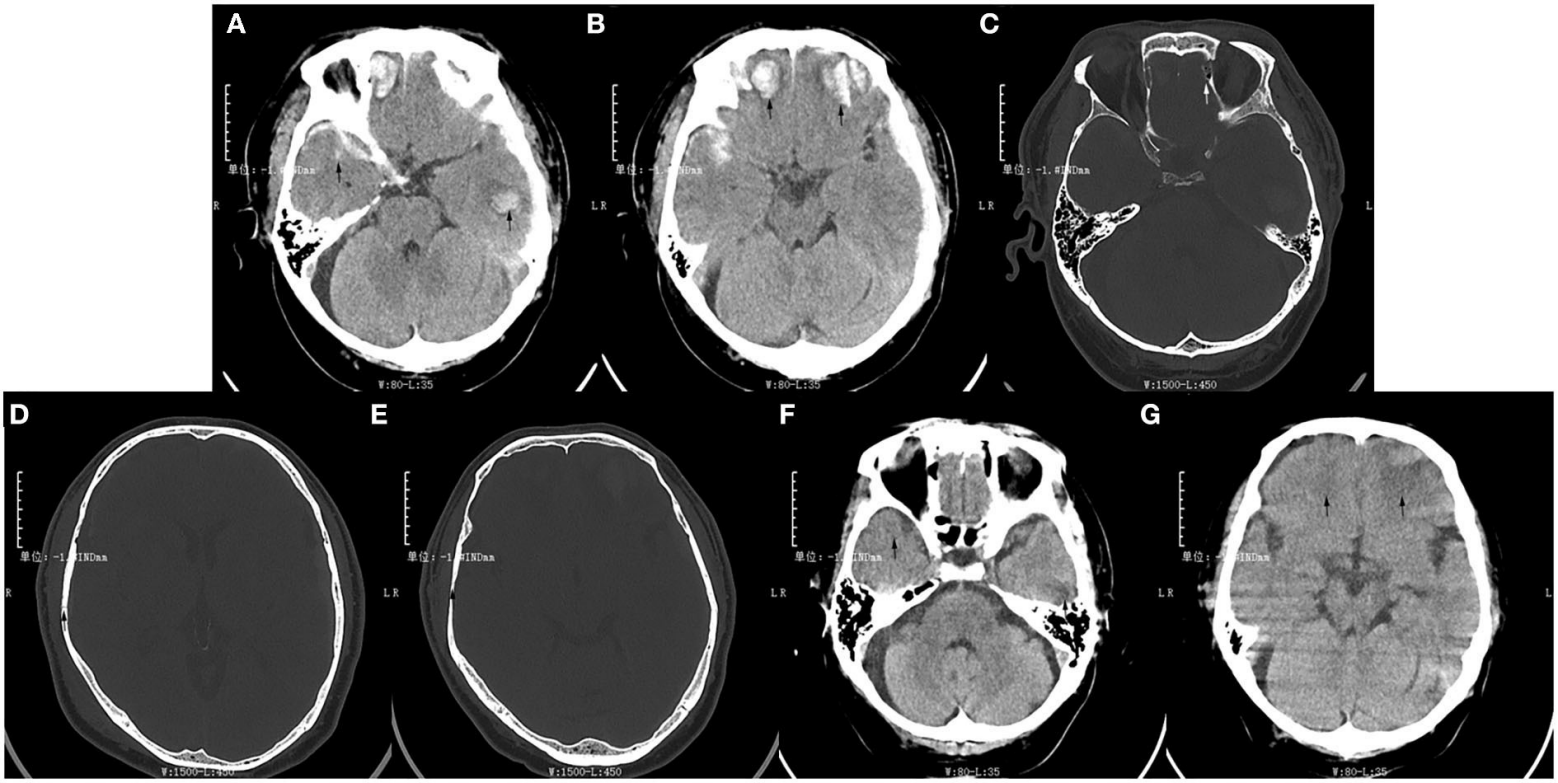
The patient's brain computed tomography results of the first hospitalization after trauma. **(A,B)** Two pieces of high-density signal were, respectively, in the bilateral temporal lobe and frontal lobe at the time of admission (arrows). **(C)** The continuity of the left orbital bone was interrupted (arrow). **(D,E)** The continuity of the right cranial parietal bone and temporal bone were interrupted (arrows). **(F,G)** The signal of lesion area displayed low-density in the bilateral temporal lobe and frontal lobe at the time of discharge (arrows).

A 53-year-old man fell down the stairs by accident and was hospitalized with TBI in the general ward of the neurology department at the Yaan Second People's Hospital. The swelling of his head and face was obvious, especially on the right side, and he had a typical left raccoon eye (Supplementary Figure 1). The brain computed tomography (CT) on admission showed two parts of abnormal increased signal in the bilateral temporal lobe and frontal lobe, and they might be confused with hemorrhage (Figures 1A,B). In addition, there was discontinuity, respectively, in the left orbital bone, the right cranial parietal bone, and the cranial temporal bone (Figures 1C–E). He was diagnosed with multiple injuries (the face, chest, and lower limbs), traumatic subarachnoid hemorrhage, bilateral frontotemporal lobe brain contusion, right temporoparietal and left orbital bone fractures, and right temporoparietal occipital scalp hematoma, right rib fracture (3–6), pulmonary contusion and multiple contusions of lower limbs. The patient was treated well-symptomatically and discharged more than 1 month later. The signal of the previous abnormal parts in the CT changed to low density, they might be manifestations of bleeding absorption (Figures 1F,G). However, pains persisted in the left forehead, especially a few days before the second hospitalization. During the previous month, the right limb occurred weakness twice, which continued for 5 min, but he did not go to hospital. Later, he complained that right limb weakness for more than 4 h and was admitted to our neurology ward at the Yaan People's Hospital. The patient's symptoms continued to worsen. Moreover, he responded slowly, and showed slurred speech, headache, and dizziness. There were no obvious symptoms of fever, dyspnea, gaze, or movement disturbance of other parts. He denied any history of hypertension, diabetes, hyperlipidemia, coronary heart disease, etc. His family had no other history of related diseases.

The physical examination revealed his blood pressure of 150/85 mmHg, and a respiratory rate of 20 breaths per minute. The general condition of the patient was fair, and he had normal auscultatory findings of his heart and lungs. The other general physical examination was normal. The neurological examination showed that his eyes had sensitive to direct and indirect light reflex. However, the right nasolabial fold became shallow, and the tongue stuck out to the right. The neck was soft, the meningeal irritation sign was negative. In the physical movement examination, he could not hold objects with the right upper limb and could not stand and walk. The muscle strength of the right limb was grade 1, and the muscle strength of the left limb was grade 5. The pathological signs of the right side were positive. He had no limbs convulsions. No other focal neurological deficits were elicited. His ability of memory, calculation, time and space orientation all descended. He refused to do the other coordination movement examinations due to inconvenience.

**Figure 2:**
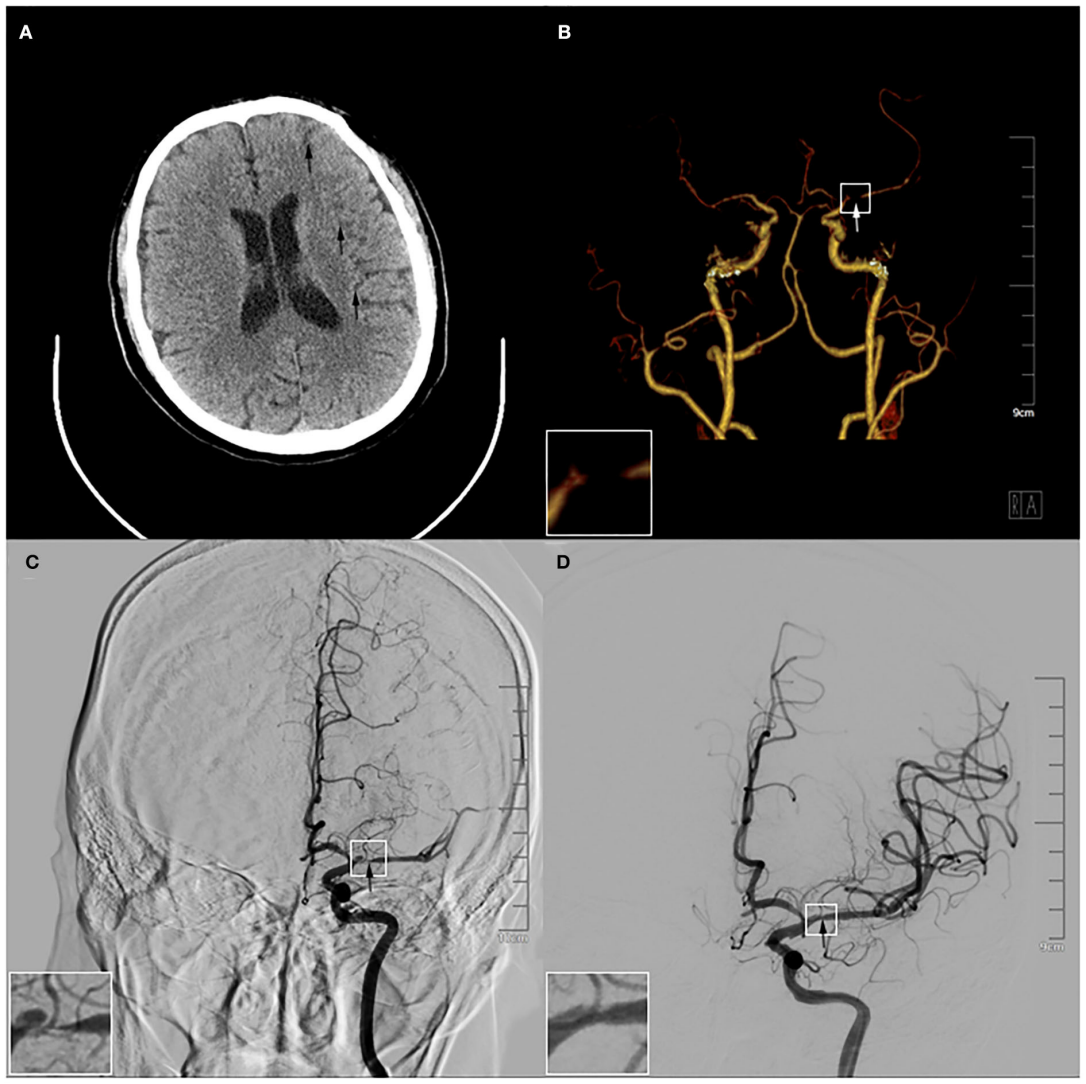
Results of preoperative and intraoperative imaging examination. **(A)** The brain computed tomography showed multiple pieces of low-density signal were in the left cerebral hemisphere (arrows). **(B)** The computed tomography angiography exhibited the M1 segment of the left middle cerebral artery (LMCA) was partially interrupted (arrow). **(C)** The angiography before the operation displayed that there was stratification in the M1 segment of the LMCA (arrow). **(D)** The angiography after the operation demonstrated the stratification disappeared after the 3.5*20 mm intracranial self-expanding stent had been released in the LMCA and the vascular morphology was significantly improved (arrow). The magnification of the white box were in the left lower corner of **(B–D)**.

In the imaging examination, the brain CT displayed multiple pieces of decreased signal in the left cerebral hemisphere, this indicated the possibility of AIS (Figure 2A). The artery and angiography exhibited that the M1 segment of the left middle cerebral artery (LMCA) was partially interrupted, and distal blood flow reduced significantly (Figure 2B). The Alberta Stroke Program Early CT Score (ASPECTS) was 8. Moreover, the value of blood glucose was 8.26 mmol/L. The other tests showed normal generally, including the blood test, the biochemical test, the urine test, the stool test, the myocardial enzyme test, and the coagulation test.

After completing various examinations, his National Institute of Health Stroke Scale (NIHSS) score was 13 and Modified Rankin Scale (MRS) score was grade 4. He had clear indications for emergency endovascular treatment and agreed with the interventional operation. The angiography showed there was dissection in the M1 segment of the LMCA (Figure 2C). The result showed that IAD was formed in the patient. He was operated on with balloon dilation and the self-expanding stent of the LMCA under general anesthesia. During the operation, the 3.5^*^20 mm intracranial self-expanding stent was released, and the LMCA was completely reanalyzed (Figure 2D). The patient was finally diagnosed with AIS and IAD (the M1 segment of the LMCA). The patient recovered well after surgery. Post-operative imaging showed no obvious abnormality, and the lesion gradually recovered (Supplementary Figures 2A,B). After 3 days, he was transferred to our general ward for rehabilitation. During treatment, he was given the aspirin tablet (100 mg once a day) and the clopidogrel tablet (75 mg once a day) to reduce the risk for progressive thrombosis, the atorvastatin tablet (20 mg once a day) to regulate lipid and stabilize plaque, the mannitol injection (125 ml once every 8 h) by intravenous and the furosemide injection (10 mg once every 12 h) to dehydrate and lower intracranial pressure. Subsequently, he requested to be discharged after the clinical symptoms were relieved (Supplementary Video 1), and his NIHSS score was 2 and MRS score was grade 0. Hereafter, we maintained telephone communication with him, and we asked him to take medication and rehabilitation on time. The patient is recovering well.

## Discussion

In our case, a man presented severe TBI to his head because of falling over 2 months ago. He recovered at home after initial treatment in hospital, then suffered intermittent headache and twice right limb weakness. These had been ignored, resulting in serious consequences. This rare but dangerous case of IAD in the BCVIs manifested in a non-specific, delayed fashion making it a challenging diagnosis for the neurophysicians to make during the initial medical encounter. In addition, the lesion of the brain was opposite to the area with serious injury at first, which may be caused by contrecoup injury. The predictability and diagnosability of the disease become complicated, and it might not be captured by standard screening criteria. Therefore, it is important to know BCVIs and IAD for improving the prognosis of these multiply injured patients.

Generally, the BCVIs are considered to be a rare entity in patients with high-energy trauma and are a potentially preventable cause of secondary brain damage. They are mainly injuries of the carotid and vertebral arteries in patients caused by head and neck blunt trauma (7), the stretching or impingement of the arteries may injure the vessel intima (3). However, the exact pathophysiological mechanism remains unclear. An analysis by Esnault et al. (2) confirmed that the main risk factors of BCVI after severe TBI were motorcycle crash, fracture involving the carotid canal, cervical spine injury, thoracic trauma, and hepatic lesion. But the patient age appeared to play a contradictory role in BCVIs risk and BCVIs-associated mortality (4). A study by Biffl et al. (5) indicated that BCVI severity may be graded using the Biffl scale, and they were I (mild intimal injury), II (dissection with raised intimal flap or intraluminal hematoma with luminal narrowing or intraluminal thrombosis), III (pseudoaneurysm), IV (vessel occlusion or thrombosis), and V (vessel transection). In our case, our patient had AIS. It was reported that ischemic stroke had been estimated to occur in up to 26% of patients with BCVIs, often within the first 72 h post-injury (6, 8). Lee's study showed the shape of intramural hematomas was independently associated with cerebral infarction. Proximal dominant intramural hematomas may be more closely associated with the cerebral infarctions than the distal dominant intramural hematomas (9). In addition, pseudoaneurysms are less common and occur because of partial transection of the artery. In general, the main risk factors are high-velocity lesions and injuries near cervical arteries. However, the main injury site of the patient in the case was the head and face, and there was no abnormality in the carotid and vertebral artery examination, which was not easily considered BCVIs.

The data on the pathophysiology, clinical, and radiological characteristics of IAD have remained limited. Traumatic IAD is difficult to diagnose in the early period of injury and is associated with high mortality. A study suggested that the high number of male patients with IAD may be related to the high number of trauma (10). The mechanisms of IAD are probably a combination of linear, torsional forces or the vessel impinges against the underlying bone. Over time, the intimal tear may cause subi-ntimal dissection of the vessel. The intimal flaps and multivessel dissections were more common after a traumatic etiology (11). Taking our case as an example, the IAD gradually formed a thrombogenic surface, platelet aggregation, and the formation of a thrombus that was partial, complete, or with secondary embolization. Therefore, the symptoms of headache progressed slowly, and the typical right limb weakness did not appear until 2 months later. There seems to be a lack of consensus regarding the optimal diagnostic strategy for the detection of IAD. On the one hand, headaches and cerebral vascular ischemic events are important references. On the other hand, DSA remains the gold standard of diagnosis. The decision to pursue vascular imaging (generally CTA) is based on the clinical and imaging findings. A recent study revealed the good performance of CTA for the detection of CAD (11). Cerebrovascular injury (CVI) was found in 44.4% of patients who underwent CTA, and the use of CTA is necessary to determine the true prevalence of CVI and optimize the use of imaging modalities (12). Several grading scales or screening criteria have been developed to guide the decision to pursue vascular imaging, as well as to recommend different treatment options for various injuries (7). However, the IAD is known to exhibit various patterns of arterial imaging features such as stenosis and dilation, and negative initial imaging results are also indicated. So, recent research on non-invasive MR had become more popular. The high-resolution cardiovascular magnetic resonance imaging (hrCMR) helped visualize and characterize IAD. It could demonstrate the distinguishing morphological features of the chronic stage of spontaneous and unruptured IAD as complete normalization, complete normalization with minimal wall changes, incomplete normalization, dissecting aneurysm, and occlusion (13). It provided a significant complementary value over DSA for the diagnosis of IAD (14). In predicting IAD changes, quantitative analysis of contrast enhancement on magnetic resonance vessel wall imaging (MR–VWI) could predict the instability of unruptured IAD (15). Chronological signal changes on T1-weighted VWI had the potential as a diagnostic imaging marker for the spontaneous healing of IAD (16). Invasive diagnostic procedures such as DSA are nowadays being replaced by the sensitive and CAD-specific sequences of MR (17). However, dissection of the cervical brain-supplying vessels is not always revealed by the imaging methods that are used to detect it. In the genetics areas, a study had shown that IAD of the MCA could be associated with the genetic background of the RNF213 p.Arg4810Lys variant (18). This was a useful supplement for the comprehensive diagnosis of IAD. Based on the special clinical symptoms, we need prompt immediate diagnostic evaluation and neurovascular imaging to Confirm IAD.

In addition, comparing the clinical presentations and imaging features of traumatic artery dissection (AD) and spontaneous AD could guide management and inform prognosis. A study of craniocervical AD by Xu et al. exhibited that the patients with minor traumatic ones were presented at a much younger age with symptoms of the neck pain compared to spontaneous ones. Patients with minor traumatic AD predominantly presented at extracranial sites with more prominent features of multiple site dissection, intramural hematoma, and long-tapering stenosis (19). However, the identification of spontaneous AD is a difficult problem. A research has demonstrated that spontaneous dissection of the internal carotid or vertebral artery was characterized by a hematoma in the vessel wall. The underlying weakness of the arterial wall may be a predisposing factor. Acute unilateral pain was the main presenting symptom (20). Identification of spontaneous and traumatic AD thus depends on the neurophysician's being aware of the symptoms and signs of the disease, so that early diagnosis can be followed by appropriate treatment.

Nowadays, some of the treatment methods for IAD were the use of intravenous thrombolysis (IVT), antithrombotic (AT) therapy, anticoagulant (AC) therapy, antiplatelet (AP) therapy, endovascular treatment (EVT), and symptomatic supportive treatment. The IVT in the AIS due to IAD seemed relatively safe (21), but the lack of relevant research was insufficient to show efficacy. After the patient's ischemic risk was determined, AT therapy may be preferred as the less-invasive first-line therapy. A study by Esnault et al. (2) showed that an AT or AC therapy was recommended to prevent the occurrence or recurrence of neurovascular events. Early identification and treatment with AT have been associated with a decreased risk of the IAD in the BCVIs. A meta-analysis study showed that both AC and AP therapy seem similarly effective in preventing ischemic stroke, but AP is better tolerated in the trauma population (22). Moreover, EVT showed favorable rates of post-treatment clinical and radiologic outcomes, coupled with low rates of treatment-related complications in preventing the occurrence of ischemic stroke (6). EVT for traumatic aneurysms had a lower mortality rate under local anesthesia. EVT in patients with carotid AD and concomitant proximal intracranial occlusion was associated with a favorable outcome (23). Besides, considering the potential risk of subsequent hemorrhagic complications by recanalization of the dissected perforator, prudent postoperative management, including strict blood pressure control, was advisable for EVT against IAD involving perforators (24). Some scholars believed that superficial temporal artery-middle cerebral artery anastomosis was an effective treatment for ischemic stroke due to dissection of the intracranial internal carotid artery with middle cerebral artery extension (25). In clinical practice, hemorrhagic IADs were unstable lesions, with a high propensity for re-bleeding (up to 40%) in the acute period. Short-term follow-up imaging in patients with these hemorrhagic lesions was important (26). In addition, traumatic subarachnoid hemorrhage (TSAH) was another life-threatening intracranial bleed disease that needed to be dealt with. The rupture site in TSAH could be difficult to locate, and injury to the MCA may be overlooked if not examined routinely (27). Early endovascular or surgical intervention for patients with IAD with SAH was recommended (28). More importantly, because of the limitations of early diagnosis, early treatment is rarely practiced clinically. In the future, a lot of basic work will be needed for the in-depth research.

## Conclusion

Overall, it was considered rare that the AIS was caused by the IAD in the BCVIs. The severe TBI, headache, and limb discomfort in convalescence may help doctors promptly recognize the possibility of the IAD in the BCVIs, which is confirmed by imaging examination. Moreover, prospective large-scale randomized studies are required to optimize the early identification of patients with IAD in the future.

## Data availability statement

The original contributions presented in the study are included in the article/Supplementary material, further inquiries can be directed to the corresponding author.

## Ethics statement

The studies involving human participants were reviewed and approved by the Yaan Local Ethical Committees. The patients/participants provided their written informed consent to participate in this study. Written informed consent was obtained from the individual(s) for the publication of any potentially identifiable images or data included in this article.

## Author contributions

BZ, JW, XH, and JZ reviewed the literature and contributed to manuscript drafting. XL and ML analyzed and interpreted the imaging findings. HZ and YY were responsible for the revision of the manuscript for important intellectual content. All authors issued final approval for the version to be submitted.

## Funding

This work was funded by the Sichuan Provincial Science and Technology Department (Grant Nos. 2019ZYZF0063 and 2020YJ0497), the Sichuan Medical Association (Grant No. Q21049), and the Key Technology Plan of Yaan City (Grant No. 21KJH0006).

## Conflict of interest

The authors declare that the research was conducted in the absence of any commercial or financial relationships that could be construed as a potential conflict of interest.

## Publisher's note

All claims expressed in this article are solely those of the authors and do not necessarily represent those of their affiliated organizations, or those of the publisher, the editors and the reviewers. Any product that may be evaluated in this article, or claim that may be made by its manufacturer, is not guaranteed or endorsed by the publisher.
