# Supplementary figures and images for: Case report: Acute ischemic stroke caused by intracranial artery dissection in a patient with skull fractures
Source: Front Neurol. 2022 Oct 24;13:963396. doi: 10.3389/fneur.2022.963396 (PMC9638089; doi:10.3389/fneur.2022.963396)

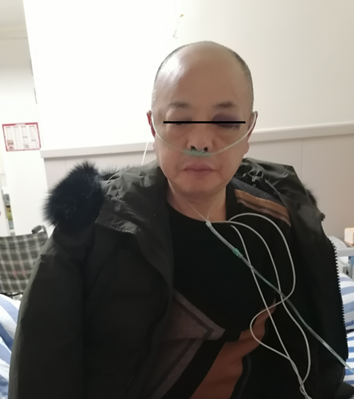

Supplement: Supplementary file 2 [file Image_1.TIF]

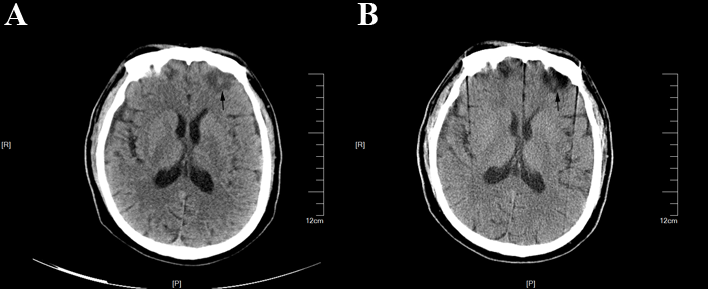

Supplement: Supplementary file 3 [file Image_2.TIF]
